# Supervised Sorting: Training Computers to Classify Toxicants

**Published:** 2004-08

**Authors:** Victoria McGovern

A new application of an existing computer learning system can improve the use of gene expression profiles to classify toxicants to which an animal has been exposed, according to work published this month by Guido Steiner and colleagues at the pharmaceutical company F. Hoffmann–La Roche **[*EHP* 112:1236–1248]**. The authors write that these investigations could help separate transcriptional changes that are of relevance for the mode of toxicity from mere bystander effects—coincidences that have little predictive value and that can be amplified by other computational approaches.

Predicting how any given toxicant will affect an organism is possible if similar compounds produce comparable changes in gene expression. Identifying useful panels of genes whose expression profiles change predictably in response to toxicants is a major goal for predictive toxicology. Finding effective ways to sort, interpret, and anticipate changes in expression is critical for moving these observations into a practical understanding of toxic effects. Background “noise” and the natural variation between experimental animals as well as compound-related characteristics in pharmacologic and toxic action complicate matters by creating variability in the analyzed data.

Other approaches to sorting gene expression data for toxicogenomics have included several “unsupervised” methods, in which modeling programs search for patterns within data and generate models of toxicity without being given any hint as to what kinds of expression patterns the researchers expect to find. The strength of this approach is that it allows for unbiased data exploration. On the other hand, it is not guaranteed to primarily retrieve information that is relevant for addressing the scientific problem at hand.

The approach reported by Steiner and colleagues is different. This group has applied a “supervised” method to toxicogenomics using what are called support vector machines (SVM). SVMs—which are computational tools, not physical machines—take advantage of additional data in the form of pathology and serum measurements that are fed into the algorithm. These data are used to assign gene expression profiles to specific modes of toxicity. The SVM identifies the most relevant information for discriminating among the given samples. After learning from a “training set” of biological samples, the model should be able to correctly classify new samples exposed to compounds that the SVM has not encountered before. Therefore, the method has to construct classification rules that still work with data different from the initial training data.

Steiner and colleagues used an SVM to find classification rules connecting patterns of gene expression in response to a series of known or suspected hepatotoxicants. The predictive genes were picked using another computational tool, recursive feature elimination (RFE), which is an integral part of SVM creation. In RFE, the computer produces a ranking of all the features that it uses to define a fingerprint—in this case, the expression profiles of each gene on a microarray. Then it calculates how much each feature contributed to that fingerprint. Uninformative or redundant features tend to be eliminated in an iterative process as less relevant, allowing refinement of the fingerprint’s definition to include only its most reliable features. These compact signatures can then be used to identify the class of toxicant to which an animal has been exposed.

In testing the system, the authors looked at 28 hepatotoxicants and 3 nonhepatotoxic compounds. Looking in rats, they laid out gene expression profiles, clinical chemistry, hematology, and histopathology for the different chemicals at various time points following exposure. In addition to discriminating between compounds that are hepatotoxic and ones that are not, their predictive models were in most cases also able to predict what kind of toxicant the animals had been exposed to—a direct-acting one that causes damage itself, a cholestatic one that interferes with bile, or a steatotic one that drives buildup of fat in the liver. By the same strategy, the SVM was able to recognize animals that had been exposed to the hepatotoxicant galactosamine but failed to respond with the typical necrosis and inflammation of the liver.

Pharmacologic activity can alter gene expression in the liver without necessarily signaling hepatotoxicity. The SVM correctly identified 3 tested pharmacoactive compounds as nonhepatotoxic and also correctly identified 3 hepatotoxic compounds whose mechanisms of toxicity were not included in the data sets used to train the machines. In two out of three cases, the SVM was able to correctly identify the general mode by which these compounds were toxic.

The models were extended to unknown rat strains, as well. After identifying expression profiles in Wistar rats induced by several peroxisome proliferator–activated receptor (PPAR) agonists, the SVM was used to look at data for the livers of Sprague-Dawley rats exposed to another PPAR agonist, WY14643. The SVM was able to correctly recognize both Sprague-Dawley rats exposed to WY14643 and the control animals, and could predict that treatment with WY14643 would stimulate peroxisome proliferation.

So far, the work has dealt predominantly with toxicant concentrations that yield substantial and largely unambiguous effects. To optimize the system to more accurately predict subtle changes, toxicologists and bioinformaticians will need both further improvements in computational methods and a larger database linking compounds and their effects on gene expression.

## Figures and Tables

**Figure:**
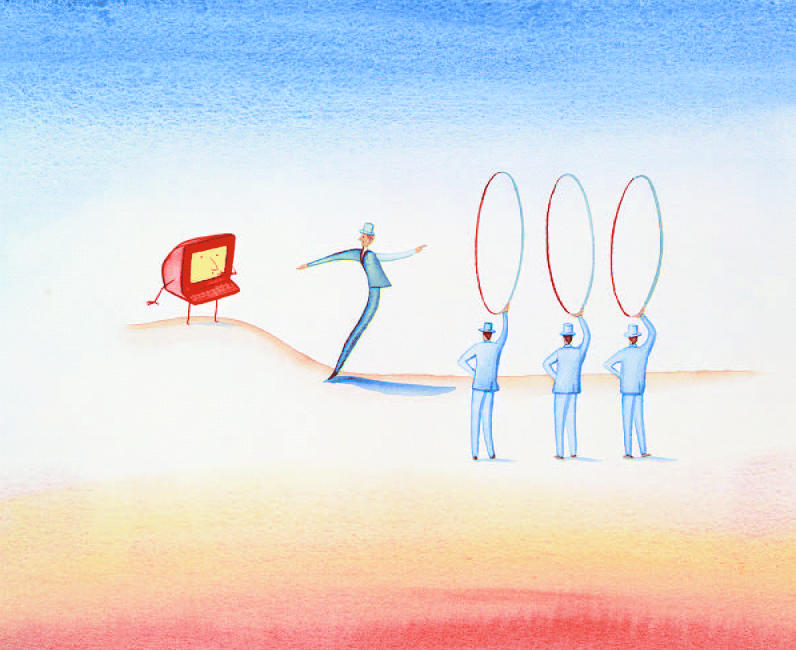
**Old computers learn new tricks.** Computational tools known as support vector machines discern relevant gene expression data from samples and apply what they learn in order to classify new compounds.

